# Supplementary material for: Synthesis of CuCo2S4@Expanded Graphite with crystal/amorphous heterointerface and defects for electromagnetic wave absorption
Source: Nat Commun. 2023 Sep 23;14:5951. doi: 10.1038/s41467-023-41697-6 (PMC10517935; doi:10.1038/s41467-023-41697-6)
Supplement: Supplementary file 1 — Supplementary Information File [file 41467_2023_41697_MOESM1_ESM.pdf]

Supplementary Information

Synthesis of $CuCo_2S_4$@Expanded Graphite with crystal/amorphous heterointerface and defects for electromagnetic wave absorption

Zhimeng Tang [a,b,d], Lei Xu [a,b,c,d,*], Cheng Xie [a,b,c], Lirong Guo [b,c,d], Libo Zhang [a,b,c], Shenghui Guo [a,b,c], Jinhui Peng [a,b,c,*]

[a] Faculty of Metallurgical and Energy Engineering, Kunming University of Science and Technology, Kunming 650093, PR China

[b] National Local Joint Laboratory of Engineering Application of Microwave Energy and Equipment Technology, Kunming University of Science and Technology, Kunming 650093, PR China

[c] The Key Laboratory of Unconventional Metallurgy, Ministry of Education, Kunming University of Science and Technology, Kunming 650093, PR China

[d] State Key Laboratory of Complex Nonferrous Metal Resources Clean Utilization, Kunming University of Science and Technology, Kunming 650093, PR China

* Corresponding author: Tel: +86 0871 65138997; Fax: +86 0871 65138997

E-mail address: Lei Xu (xu_lei@kust.edu.cn) Jinhui Peng (jhpeng@kust.edu.cn)

Supplementary equations

(1) Magnetic loss of composite materials is generally caused by natural resonance, exchange resonance and eddy current effect. In order to further analyze the type of magnetic loss, C_0 is introduced and the equation is as follows:

$$C_0 = \mu''(\mu')^{-2}f^{-1} = 2\pi\mu_0\sigma d^2/3 \quad (1)$$

The μ_0 for vacuum magnetic permeability, σ for conductivity and f frequency.

(2) According to the theory of debye ε'' depends on the conduction loss (ε_c'') and polarization loss (ε_p'') comprehensive contribution. Its equation is as follows:

$$\varepsilon'' = \frac{\varepsilon_s-\varepsilon_\infty}{1+\omega^2\tau^2}\omega\tau + \frac{\sigma}{\omega\varepsilon_0} = \varepsilon_p'' + \varepsilon_c'' \quad (2)$$

$$\varepsilon_c'' = \frac{\sigma}{\omega\varepsilon_0} \quad (3)$$

$$\varepsilon_p'' = \frac{\varepsilon_s-\varepsilon_\infty}{1+\omega^2\tau^2}\omega\tau = \varepsilon'' - \varepsilon_c'' \quad (4)$$

Among them, the ω for angular frequency, σ for electrical conductivity, ε_0 for the vacuum dielectric constant (8.85×10^{-12} Fm^{-1}), ε_s as the static dielectric constant, ε_∞ for infinite frequency dielectric constant, τ as relaxation time.

(3) In order to study the polarization relaxation process, uses the ε' - ε'' curve to describe the cole-cole semicircle. According to the theory of Debye ε' and ε'' relationship can be expressed in the type.

$$\left(\varepsilon' - \frac{\varepsilon_s+\varepsilon_\infty}{2}\right)^2 + \left(\varepsilon''\right)^2 = \left(\frac{\varepsilon_s-\varepsilon_\infty}{2}\right)^2 \quad (5)$$

(4) The normalized input impedance (Z) is expressed by the equation, as following:

$$Z = \left|\frac{Z_{in}}{Z_0}\right| = \sqrt{\frac{\mu_r}{\varepsilon_r}}tanh\left[j(\frac{2\pi fd}{c})\sqrt{\mu_r\varepsilon_r}\right] \quad (6)$$

Where, Z_{in} is the input characteristic impedance, Z_o is the free space impedance, and d is the material thickness.

Supplementary Figure 1-31

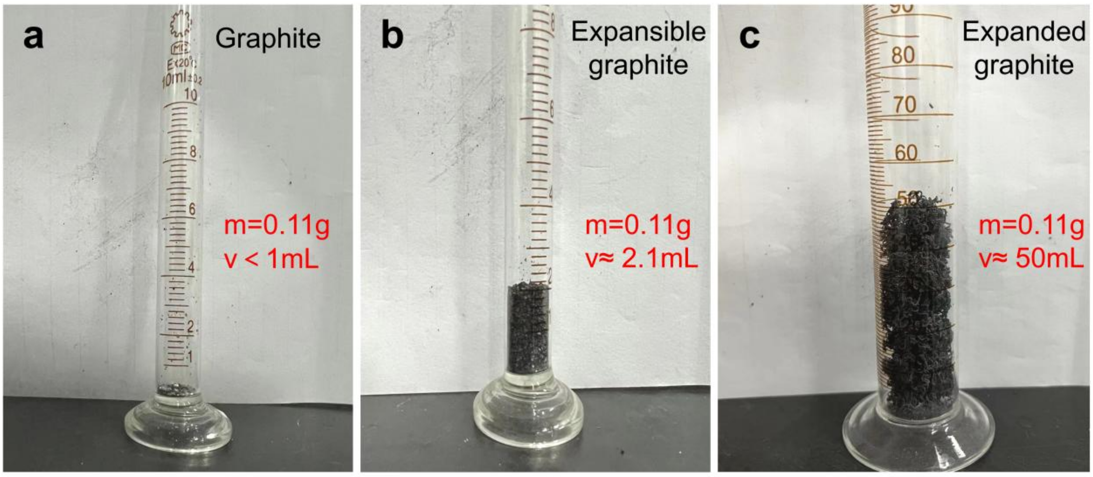

Supplementary Figure 1. Volume change of EG before and after microwave treatment. a Volume of graphite. **b** Volume of expansible graphite. **c** Volume of expanded graphite.

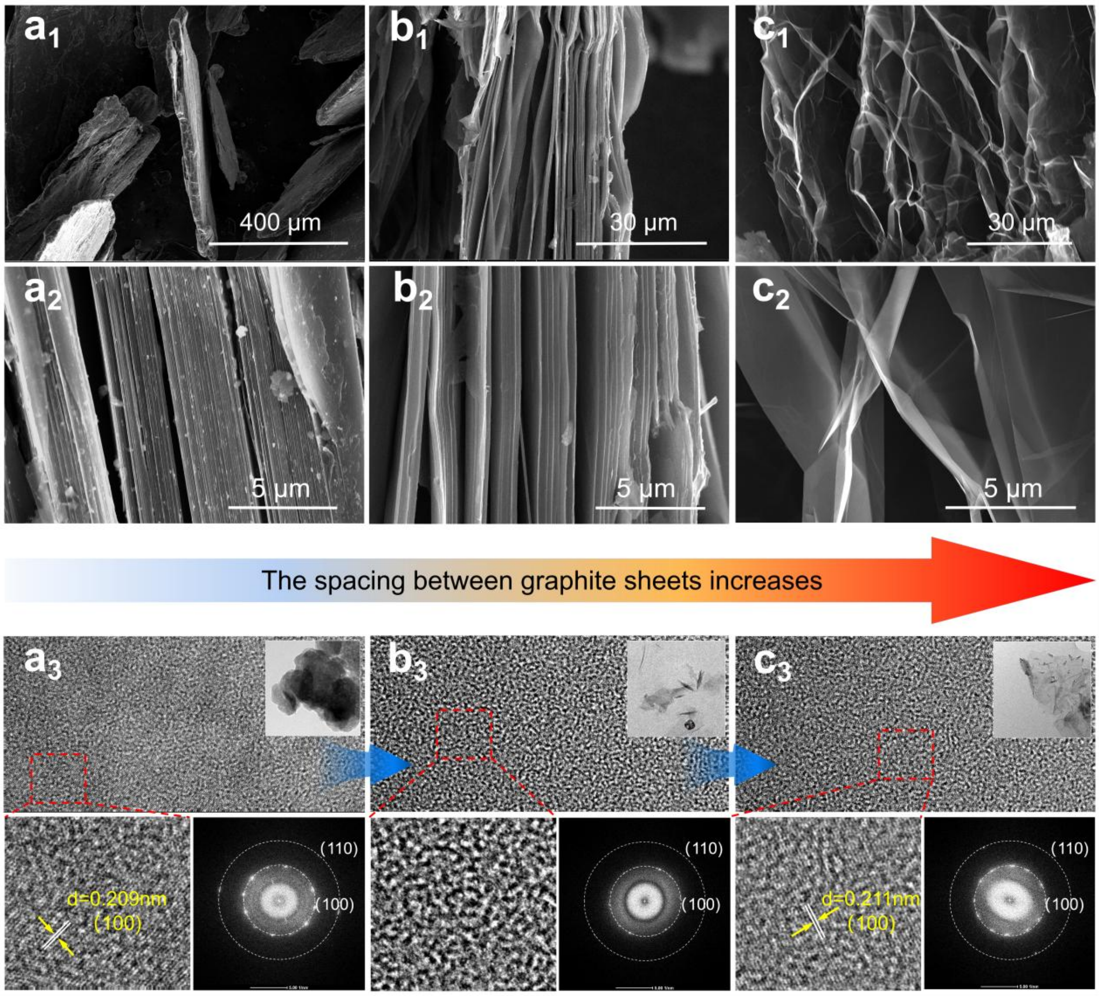

Supplementary Figure 2. Microscopic morphology of EG before and after microwave treatment. $\mathbf{a_1}$, $\mathbf{a_2}$, $\mathbf{a_3}$ SEM and TEM images of graphite. $\mathbf{b_1}$, $\mathbf{b_2}$, $\mathbf{b_3}$ SEM and TEM images of expansible graphite. $\mathbf{c_1}$, $\mathbf{c_2}$, $\mathbf{c_3}$ SEM and TEM images of expanded graphite.

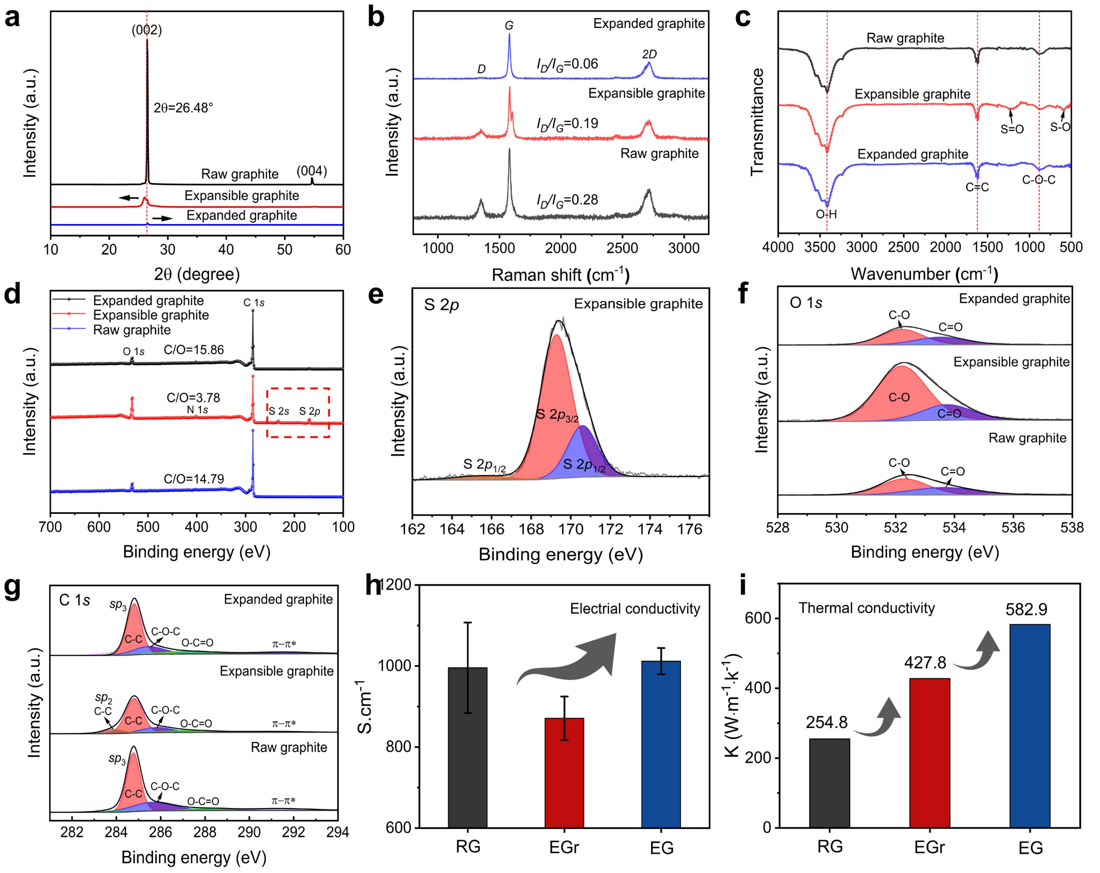

Supplementary Figure 3. Structural and physical characteristics of EG before and after microwave treatment were measured. **a** XRD patterns of raw graphite, expansible graphite and expanded graphite. **b** Raman spectra of raw graphite, expansible graphite and expanded graphite. **c** FTIR spectra of raw graphite, expansible graphite and expanded graphite. **d**, **e**, **f**, **g** XPS spectra of survey, S 2*p*, C 1*s* and O 1*s* of raw graphite, expansible graphite and expanded graphite. **h** Electrical conductivity of RG, EGr and EG, the error bars represent standard deviations were estimated by two experiments. **i** Thermal conductivity of RG, EGr and EG.

Supplementary Figure 3a shows the X-ray diffraction results before and after EG treatment. The strong diffraction characteristic peaks of graphite at (002) and (004) are clearly observed in the Figure. However, after intercalation and microwave

treatment, the diffraction intensity of graphite peak decreased significantly, and the expanded graphite peak shifted to the right. Supplementary Figure 3b shows the Raman spectrum and the intensity ratio of D band to G band (I_D/I_G) of EG before and after microwave-assisted preparation. It can be observed from the Figure that there are two obvious strong absorption peaks G and 2D at 1581cm^{-1} and 2720 cm^{-1}, respectively. This indicates that EG before and after microwave-assisted treatment has very regular structure and large graphene domain size. Meanwhile, the D peak at 1338cm^{-1} was observed in the Raman spectrum. The D-peak of microwave-assisted EG disappeared almost completely, and the ratio of D-peak to G-peak was only 0.06. This is significantly different from EG prepared by the conventional method and Humer method. The intercalator and functional group change information before and after EG preparation were characterized by FTIR, as shown in Supplementary Figure 3c. In all samples, the absorption band at 3430cm^{-1} was attributed to stretching vibrations of -OH. The band at 1620cm^{-1} corresponds to C=C vibration. The band at 875cm^{-1} is attributed to C-O-C symmetry and stretching. However, new vibration peaks appear at 1240-1257cm^{-1} and 583-589cm^{-1} of expandable graphite. This is caused by the asymmetric stretching vibration of S=O and S-O. The results show that sulfuric acid and sulfate can be intercalated into graphite quickly under the condition that the graphite is not seriously caused by microwave.

In addition, XPS was used to further detect the surface chemical composition or surface functional groups of expanded graphite, as shown in Supplementary Figure 3d-g. In the range of 100eV~700eV, EG mainly shows characteristic peaks related to

C 1*s*, O 1*s* and S 2*p*. After microwave treatment, the C/O ratio of graphite decreased from 14.79 to 3.78 and then increased to 15.86. The results show that microwave irradiation can effectively reduce the C-O, C=O oxygen-containing groups covered by expanded graphite surface, which is beneficial to obtain high-performance three-dimensional carbon-based conductive network. As shown in Supplementary Figure 3h and i, the prepared expanded graphite has good thermal and electrical conductivity. This provides a new route and raw material supply for the preparation of high-performance carbon based microwave absorbing materials.

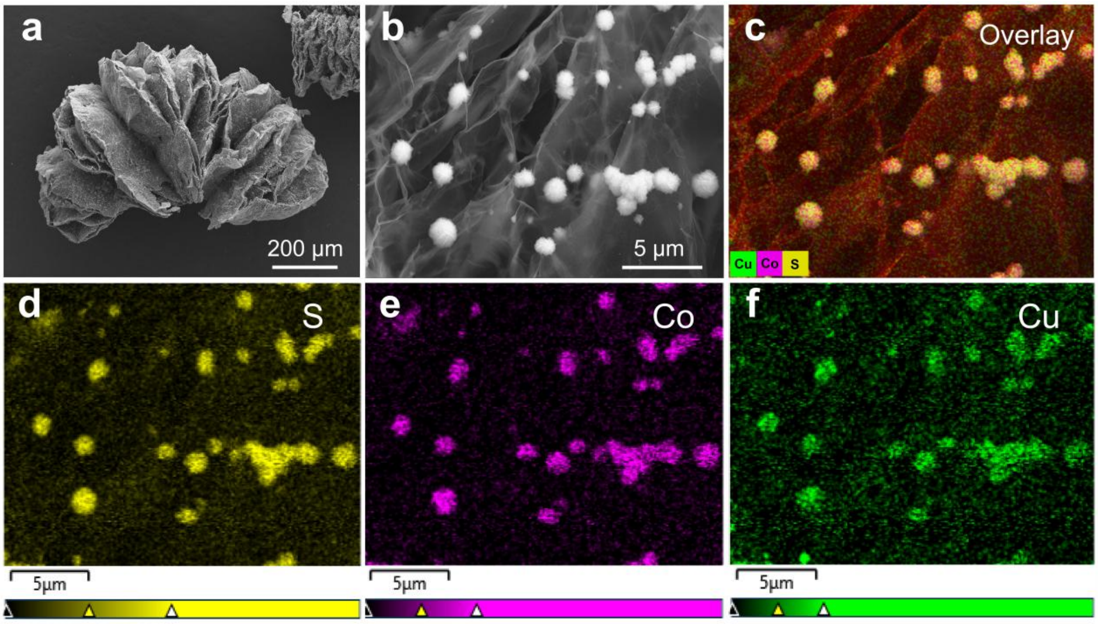

Supplementary Figure 4. The microscopic morphology of $CuCo_2S_4$@EG. a, **b** SEM images of $CuCo_2S_4$@EG. **c**, **d**, **e**, **f** Corresponding element distribution of $CuCo_2S_4$@EG. Gradient color scale shows the degree of enrichment of the element.

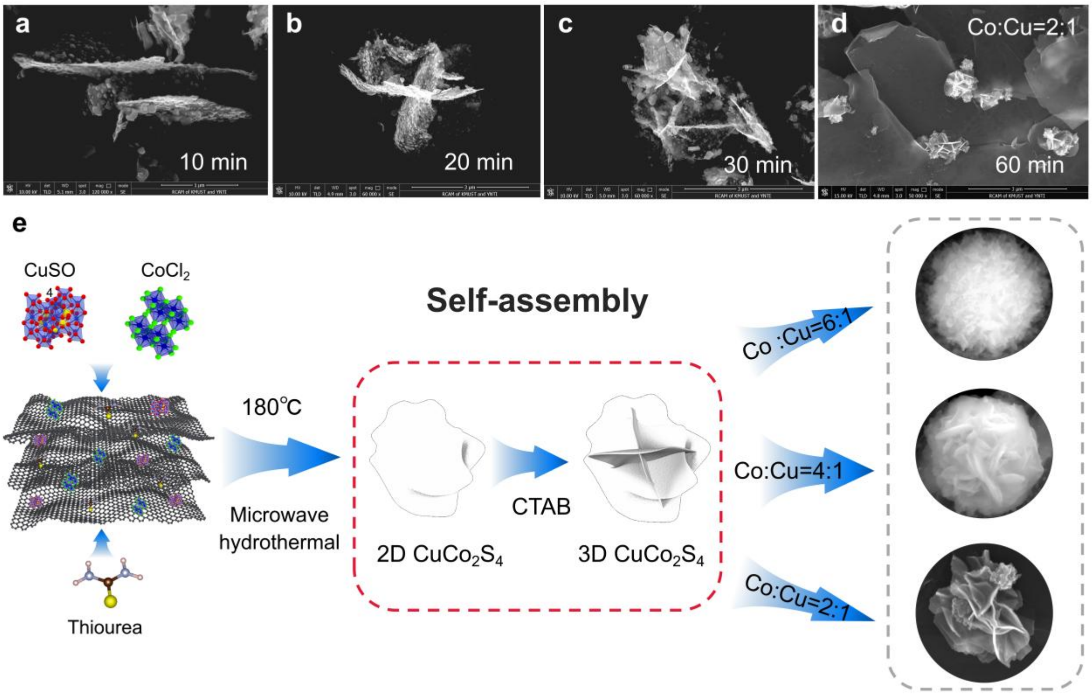

Supplementary Figure 5. Growth mechanism diagram of $CuCo_2S_4$ flower. a, **b**, **c**, **d** Microscopic morphology of $CuCo_2S_4$ under different time. **e** Growth mechanism of $CuCo_2S_4$.

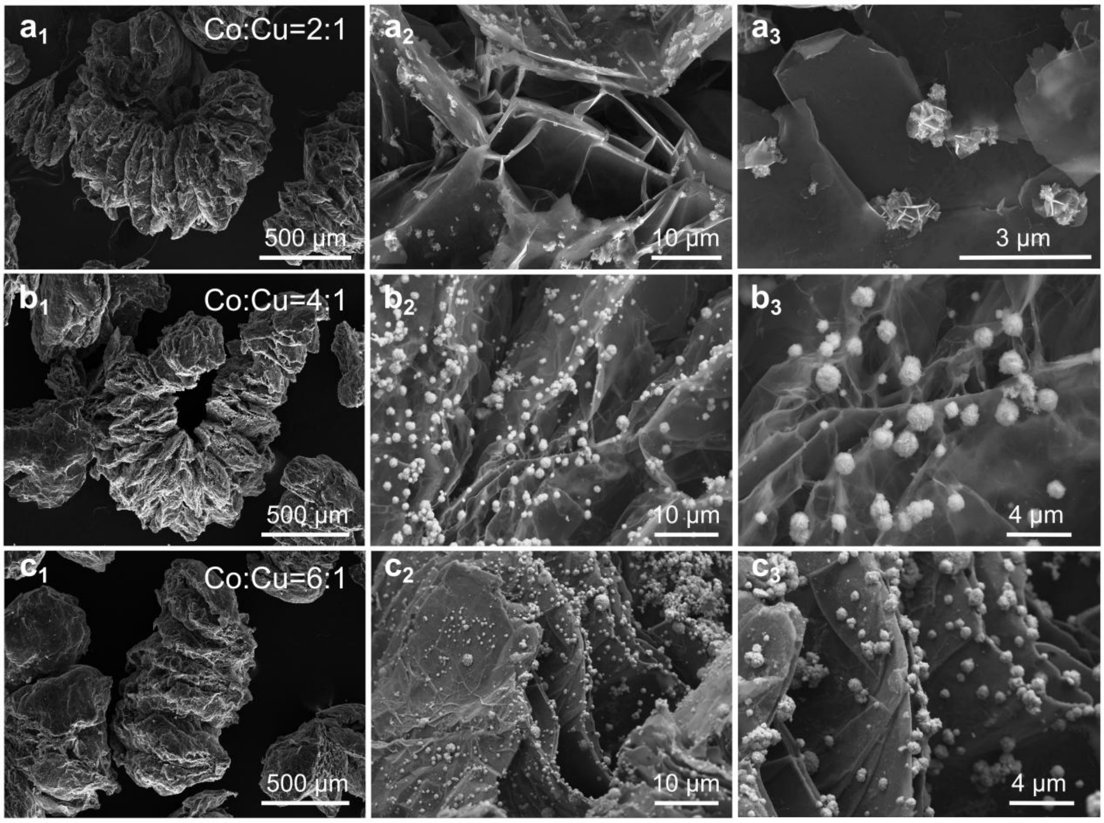

Supplementary Figure 6. The microscopic morphology of CEG-2, CEG-4 and CEG-6. **a_1**, **a_2**, **a_3** SEM images of CEG-2 at different magnifications. b_1, **b_2**, **b_3** SEM images of CEG-4 at different magnifications. **c_1**, **c_2**, **c_3** SEM images of CEG-6 at different magnifications.

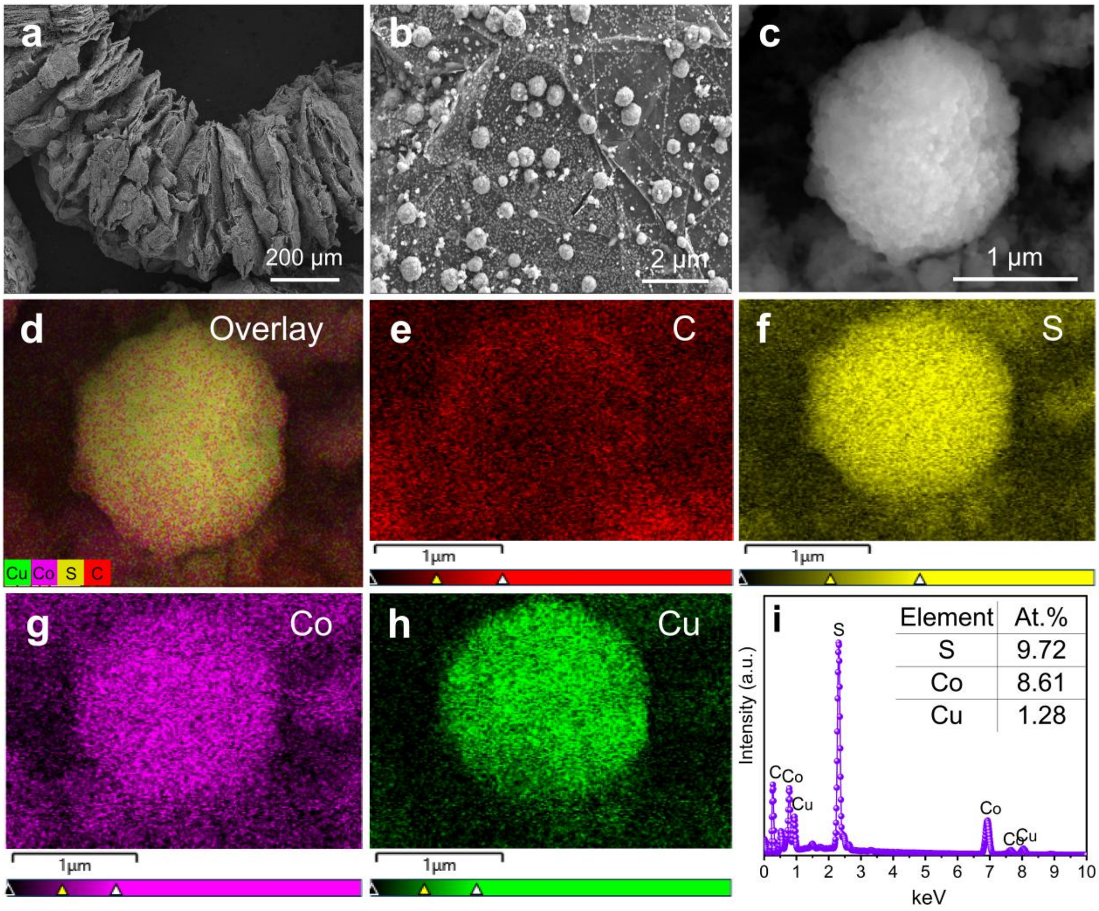

Supplementary Figure 7. The microscopic morphology of CEG-8. a, **b**, **c** SEM images of CEG-8 at different magnifications. **d**, **e**, **f**, **g**, **h** Corresponding element distribution of CEG-8, gradient color scale Shows the degree of enrichment of the element. **i** EDS spectra of the CEG-8.

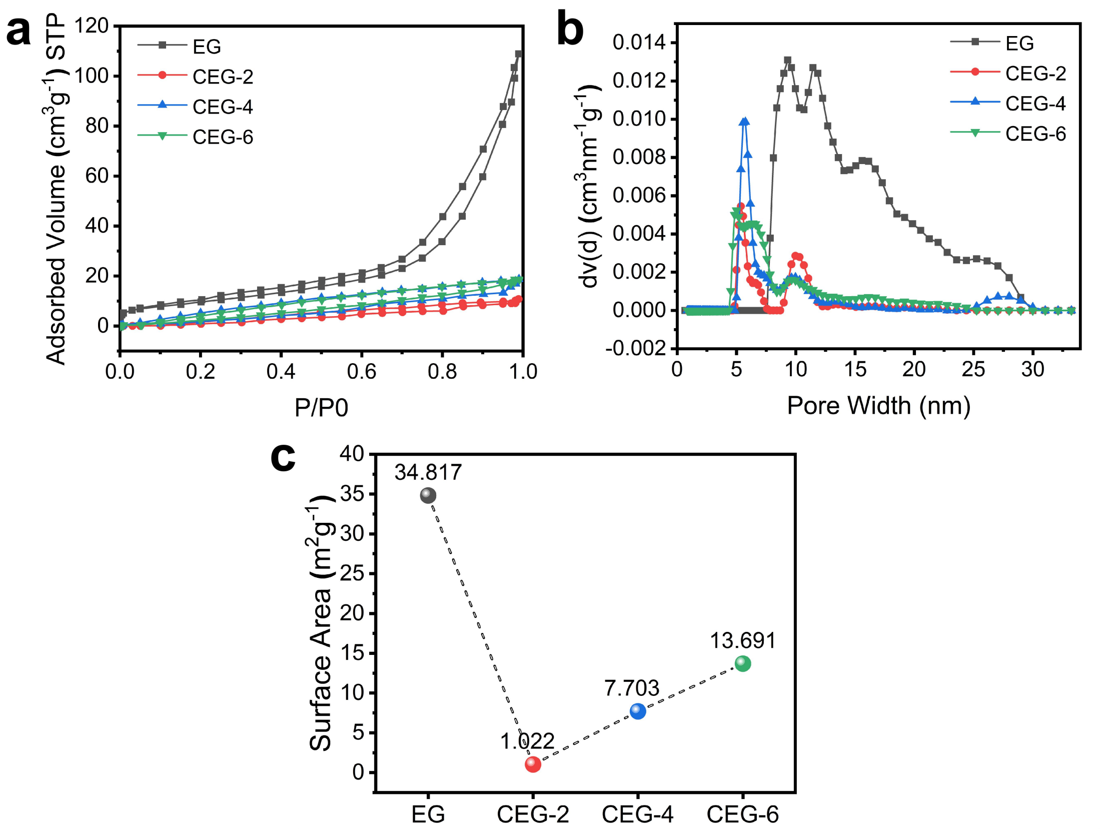

Supplementary Figure 8. The specific surface area and pore size distribution of CEG composites. a N_2 adsorption-desorption isotherms of EG, CEG-2, CEG-4 and CEG-6. **b** Pore size distribution of EG, CEG-2, CEG-4 and CEG-6. **c** Specific surface area of EG, CEG-2, CEG-4 and CEG-6.

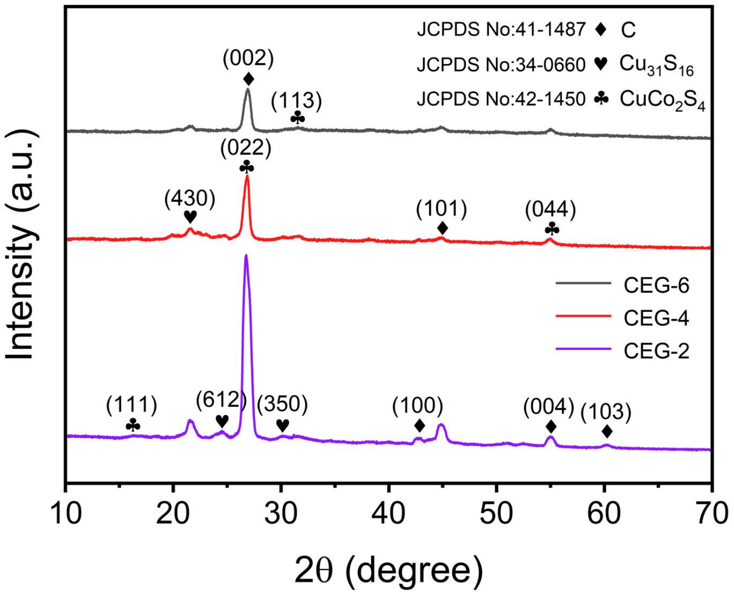

Supplementary Figure 9. XRD patterns for CEG-2, CEG-4 and CEG-6.

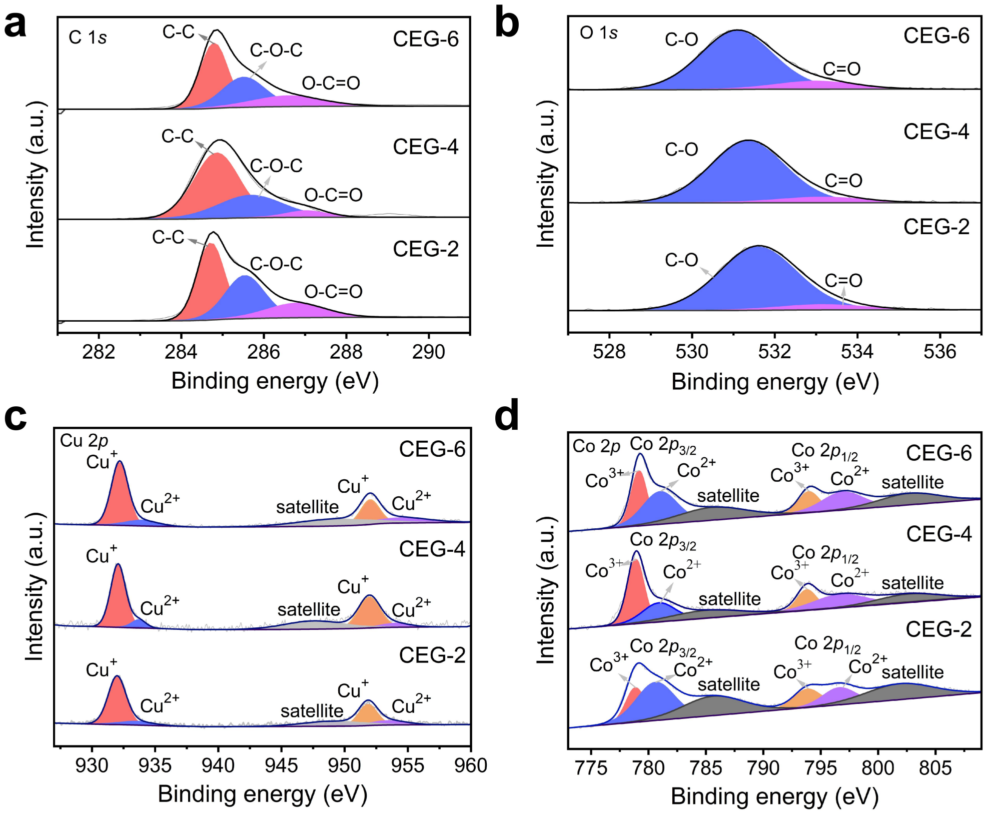

Supplementary Figure 10. The molecular structure and chemical state of CEG heterostructure surface. a, **b**, **c**, **d** XPS spectra of C 1*s*, O 1*s*, Cu 2*p* and Co 2*p* of CEG-2, CEG-4 and CEG-6.

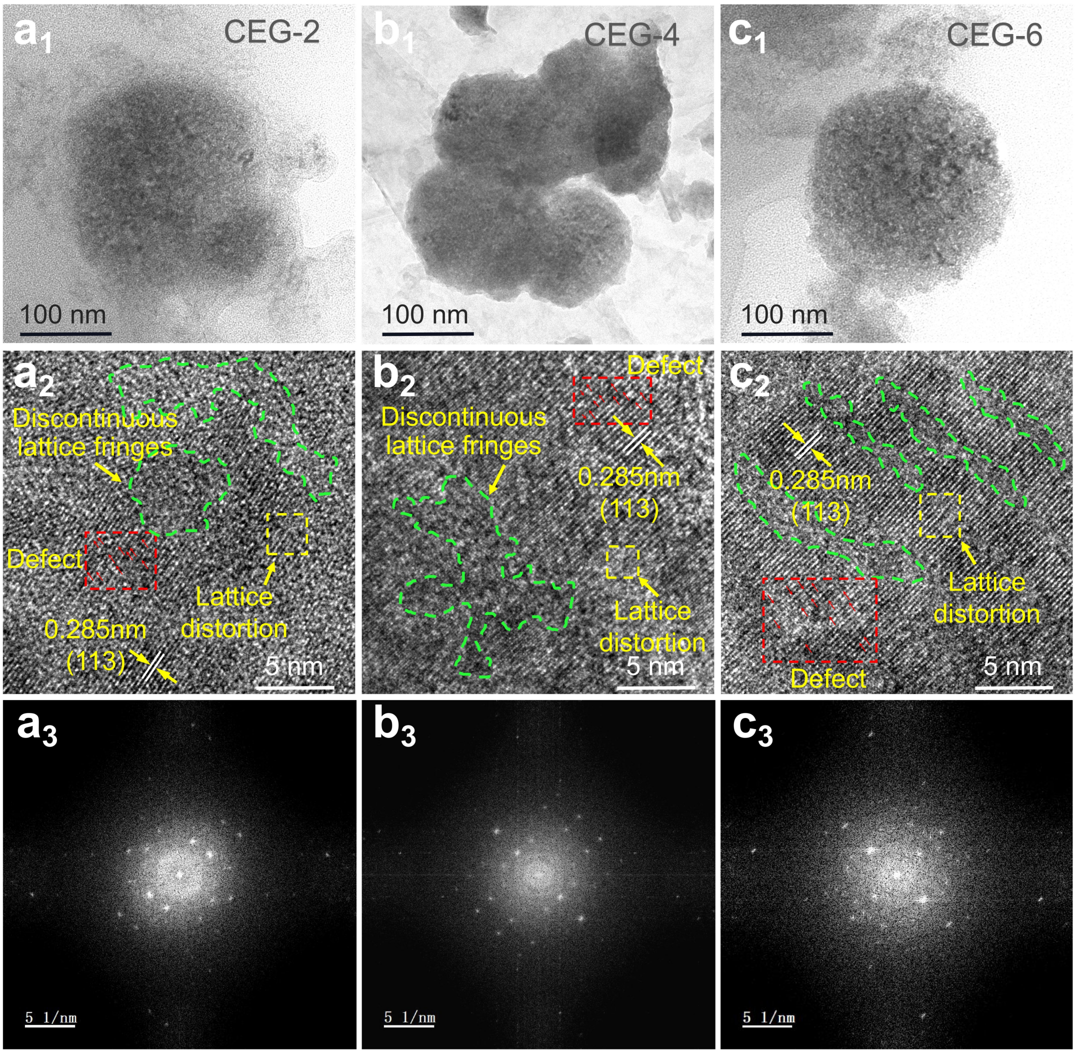

Supplementary Figure 11. HRTEM patterns and electron diffraction patterns of CEG. **a_1, a_2, a_3** TEM images of CEG-2, CEG-4 and CEG-6. **b_1, b_2, b_3** TEM images of CEG-4. **c_1, c_2, c_3** TEM images of CEG-6. The dashed green areas represent discontinuous lattice fringes, the dashed yellow areas represent lattice distortion, and the red arrow areas represent defect locations.

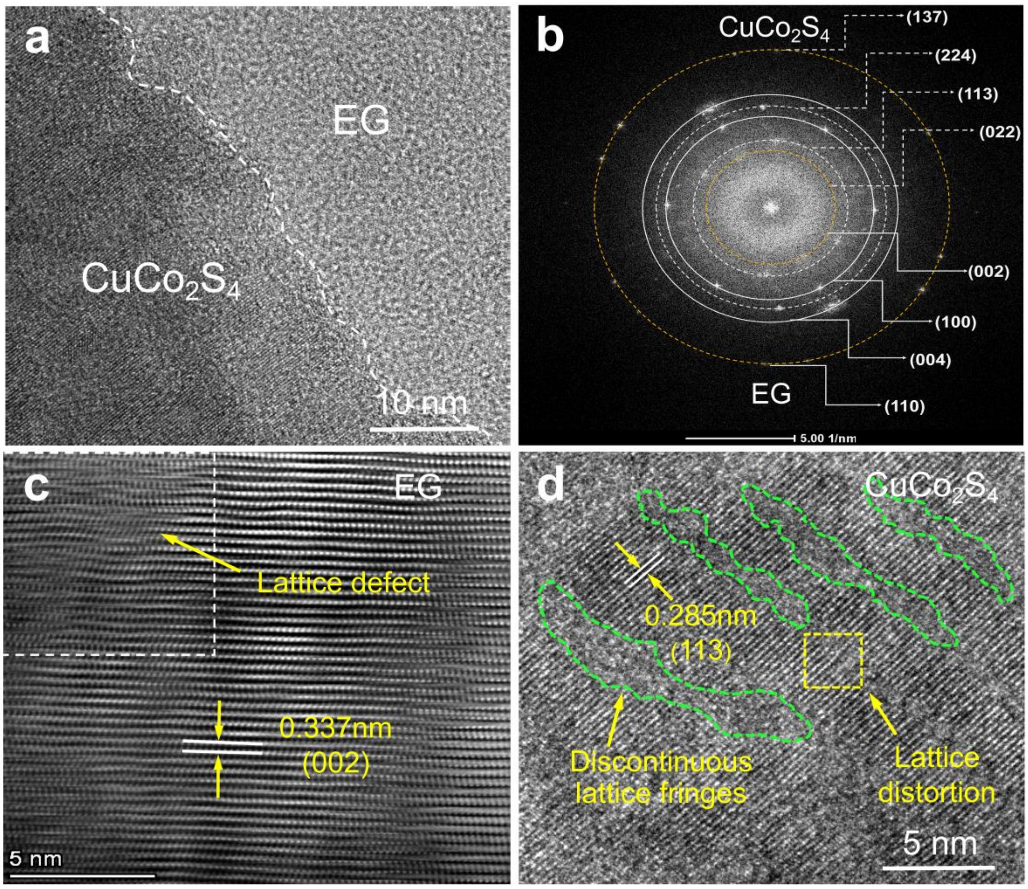

Supplementary Figure 12. The detailed microstructure and lattice information of the CEG. a-b HRTEM image of CEG-6. **c** AC HAADF-STEM image of EG, the dashed white areas represent lattice defect. **d** HRTEM images of $CuCo_2S_4$, the dashed green areas represent discontinuous lattice fringes, the dashed yellow areas represent lattice distortion.

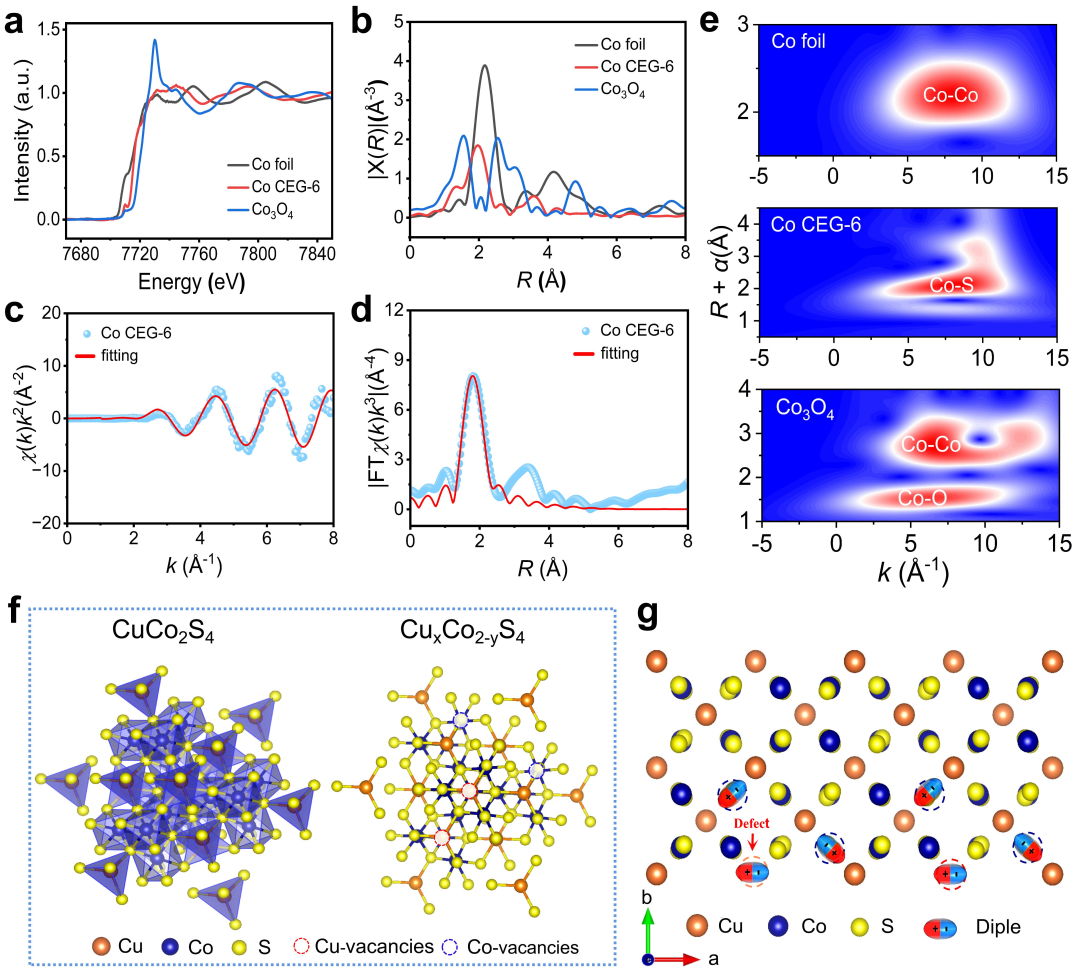

Supplementary Figure 13. Coordination environment of Co atoms. **a** Normalized XANES spectra at the Co K-edge of the Co foil, CEG-6 and Co_3O_4. **b** FT-EXAFS spectra of the Co foil, CEG-6 and Co_3O_4. **c** EXAFS fitting curve for Co CEG-6 in the k-space. **d** EXAFS fitting curve for Co CEG-6 in the R-space. **e** WT of the Co foil, CEG-6 and Co_3O_4. **f**, **g** Schematic diagram of metal cation vacancy structure of $CuCo_2S_4$.

DFT Calculations

All the calculations are performed in the framework of the density functional theory with the projector augmented plane-wave method, as implemented in the Vienna ab initio simulation package [1]. The generalzied gradient approximation proposed by Perdew, Burke, and Ernzerh of is selected for the exchange-correlation potential [2]. The long range van der Waals interaction is described by the DFT-D3 approach [3]. The cut-off energy for plane wave is set to 420 eV. The energy criterion is set to 10^{-6} eV in iterative solution of the Kohn-Sham equation. The Brillouin zone integration is performed at the Gamma point with k-mesh grid of 3×3×1. All the structures are relaxed until the residual forces on the atoms have declined to less than 0.05 eV/Å.

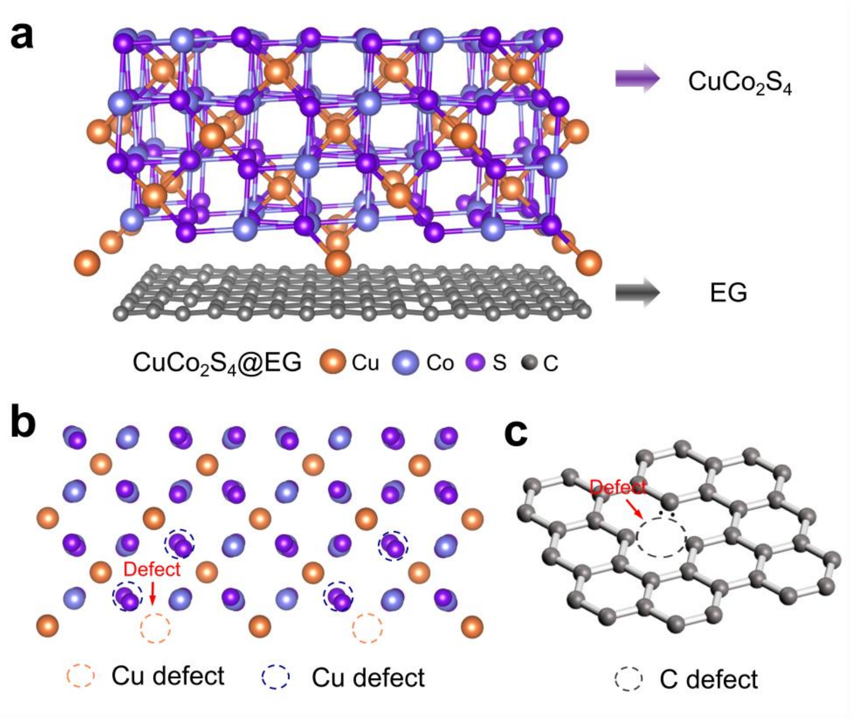

Supplementary Figure 14. Model of sample. a The model of $CuCo_2S_4$ and carbon layer. **b** The defect model of $CuCo_2S_4$. **c** The defect model of graphite.

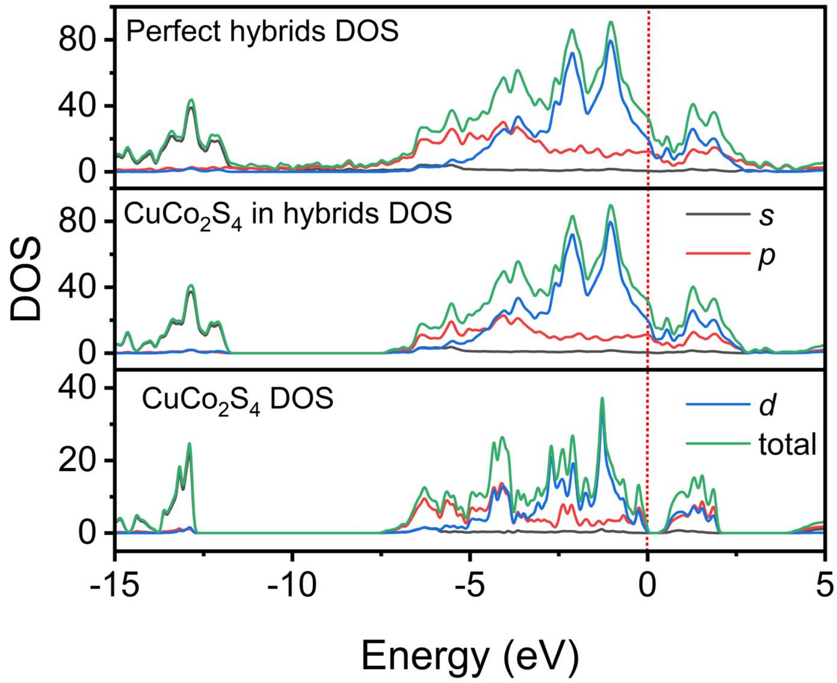

Supplementary Figure 15. Calculated DOS of the perfect CEG and $CuCo_2S_4$ structures. Red dashed line is Fermi level.

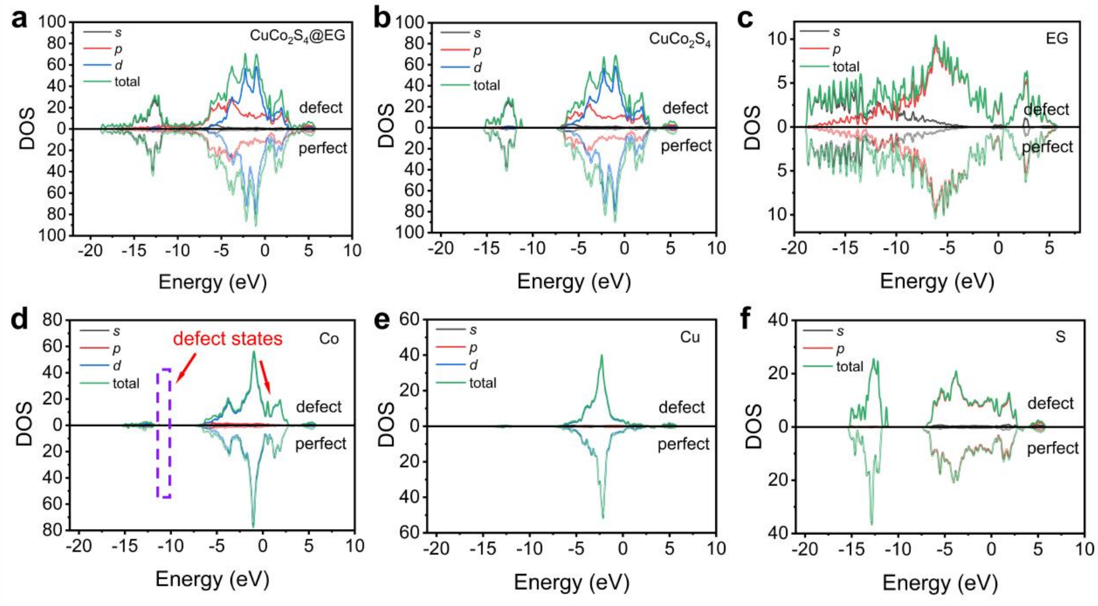

Supplementary Figure 16. The density of states (DOS) and partial density of states (PDOS) for CEG. a The DOS of the CEG heterostructure. **b** The DOS of the $CuCo_2S_4$. **c** The DOS of the EG. **d**, **e**, **f** The PDOS of S, Cu and Co elements.

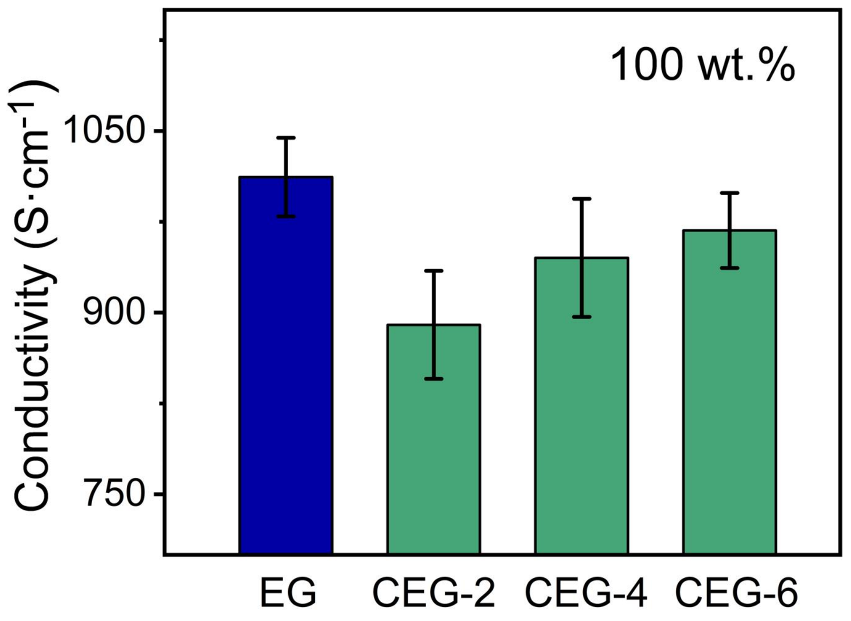

Supplementary Figure 17. Conductivity of the EG, CEG-2, CEG-4 and CEG-6 with 100 wt% filler loading, the error bars represent standard deviations were estimated by two experiments.

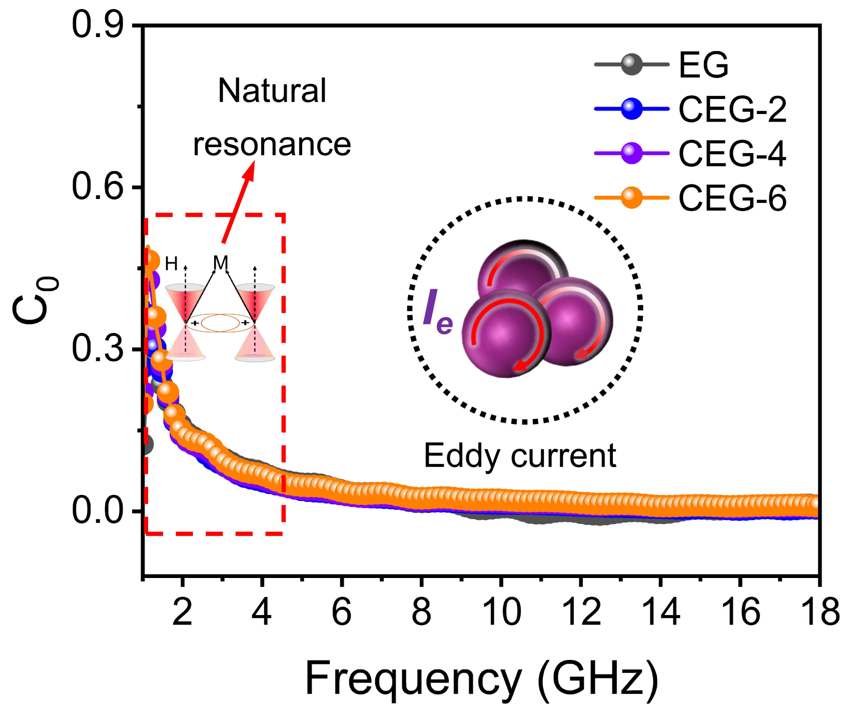

Supplementary Figure 18. Eddy current loss of EG, CEG-2, CEG-4 and CEG-6.

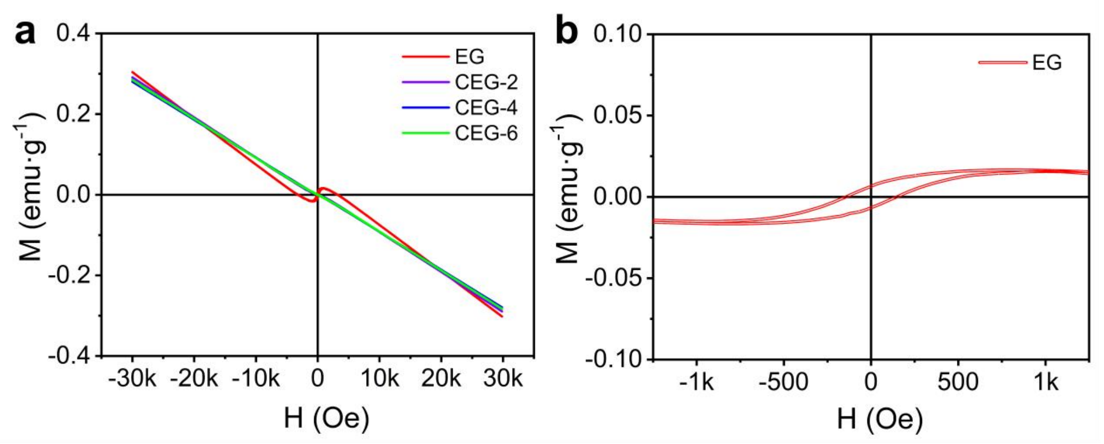

Supplementary Figure 19. Magnetic properties testing. **a** The hysteresis loop of EG, CEG-2, CEG-4 and CEG-6. **b** The hysteresis loop of EG.

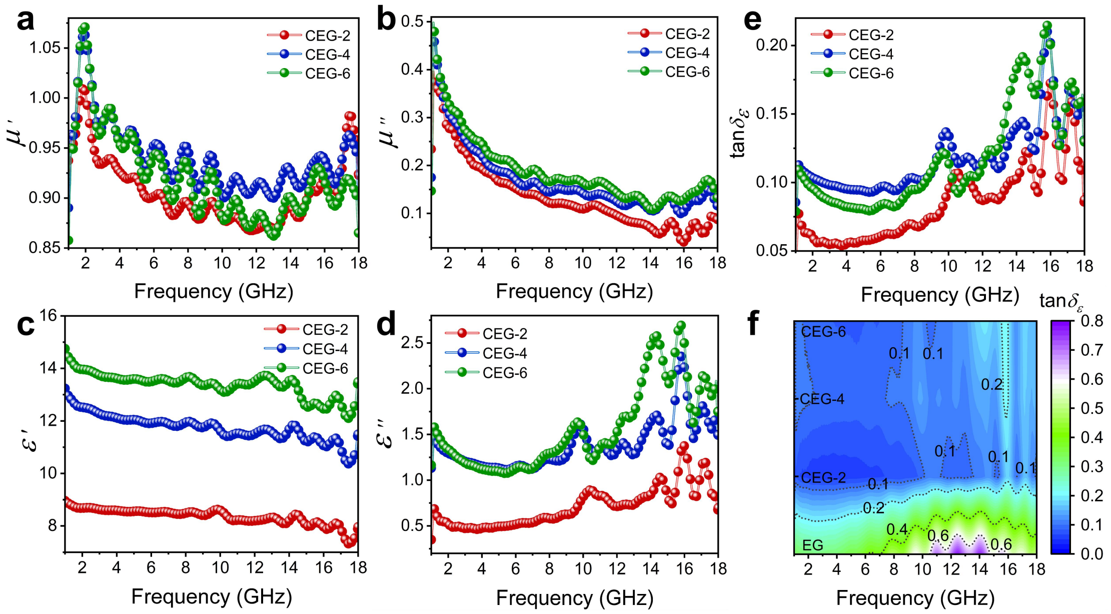

Supplementary Figure 20. Electromagnetic wave absorption properties of $CuCo_2S_4$@EG. a, **b** Real part and imaginary part of complex permeability of CEG-2, CEG-4 and CEG-6. **c, d** Real part and imaginary part of complex permittivity of CEG-2, CEG-4 and CEG-6. **e, f** Dielectric loss tangent ($\tan\delta_{\varepsilon}$) of CEG-2, CEG-4 and CEG-6.

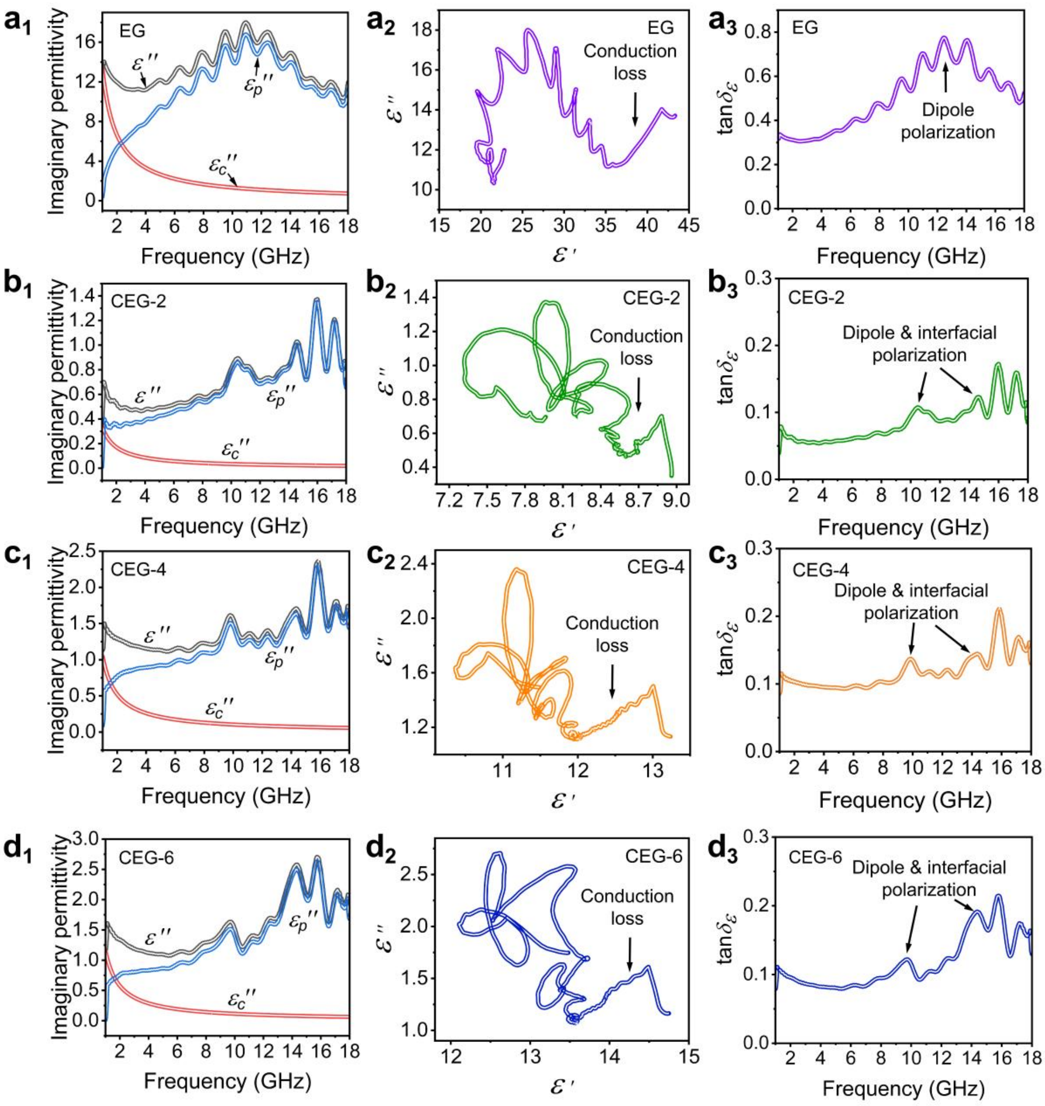

Supplementary Figure 21. Dielectric loss of the samples and Cole-Cole plots. a$_1$, b$_1$, c$_1$, d$_1$ Conduction loss ε_c'' and polarization loss ε_p'' of EG, CEG-2, CEG-4 and CEG-6. **a$_2$, b$_2$, c$_2$, d$_2$** Cole-Cole plots of EG, CEG-2, CEG-4 and CEG-6. **a$_3$, b$_3$, c$_3$, d$_3$** $tan\,\delta_\varepsilon$ of EG, CEG-2, CEG-4 and CEG-6.

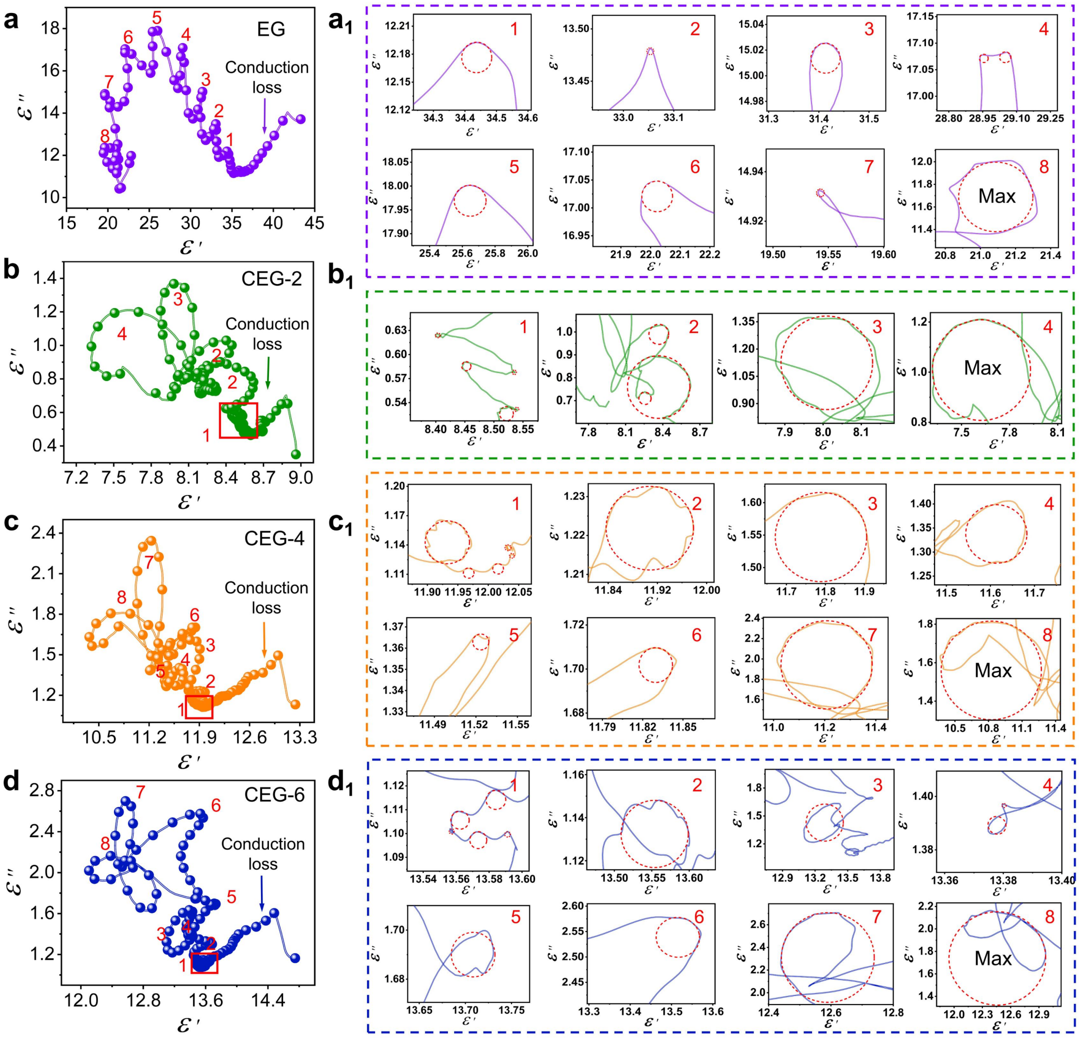

Supplementary Figure 22. Cole-Cole plots of samples. a, a$_1$ Cole-Cole plots of EG. **b, b$_1$** Cole-Cole plots of CEG-2. **c, c$_1$** Cole-Cole plots of CEG-4. **d, d$_1$** Cole-Cole plots of CEG-6.

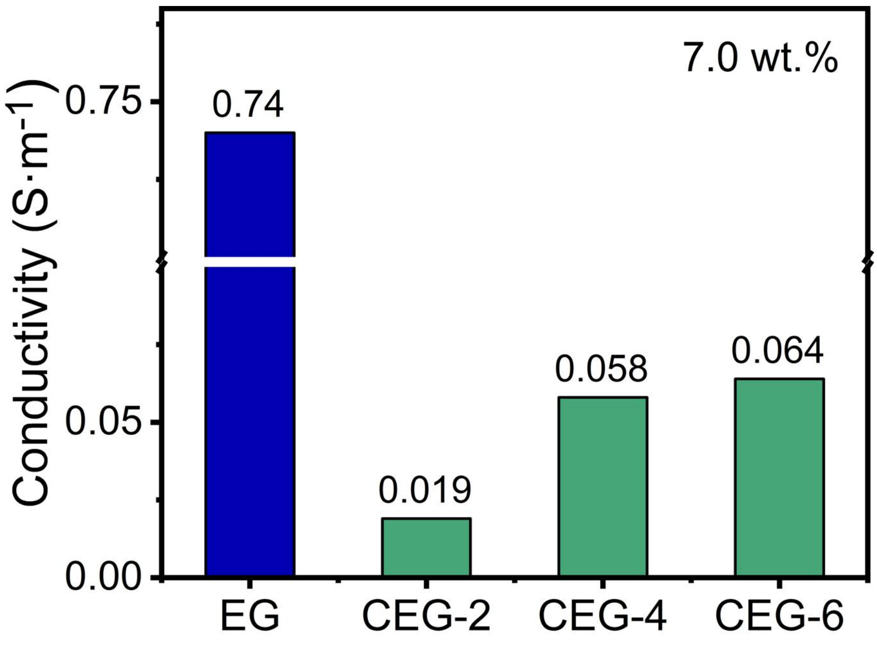

Supplementary Figure 23. DC conductivity of EG, CEG-2, CEG-4 and CEG-6 with 7.0 wt% filler loading.

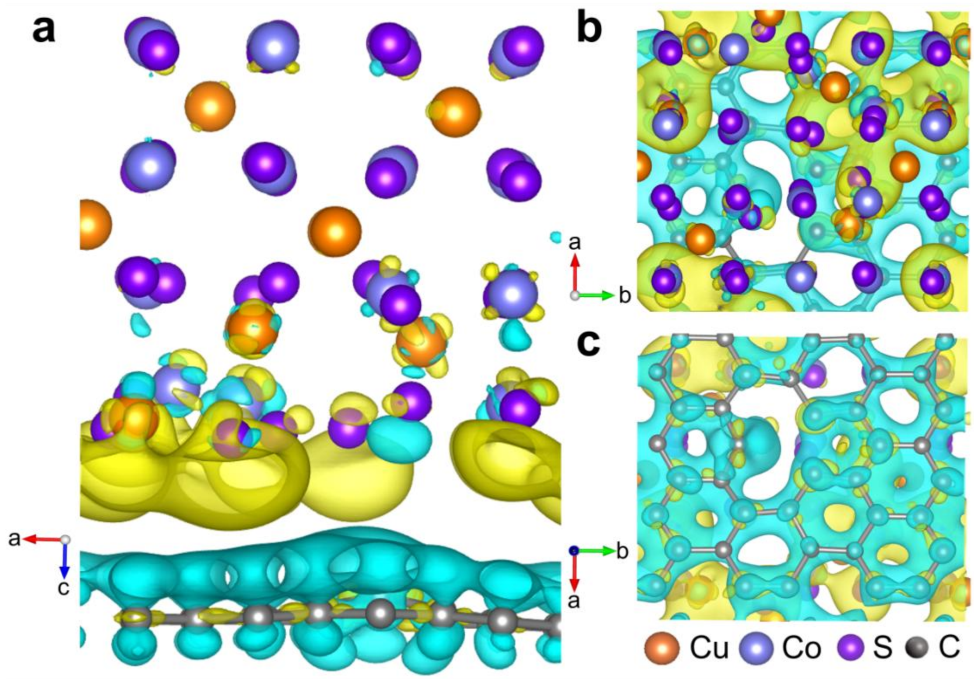

Supplementary Figure 24. Charge density difference of heterointerface. **a**, **b**, **c** Charge density difference of $CuCo_2S_4$@EG in different directions. Blue-green color represents charge depletion, while yellow color represents charge accumulation.

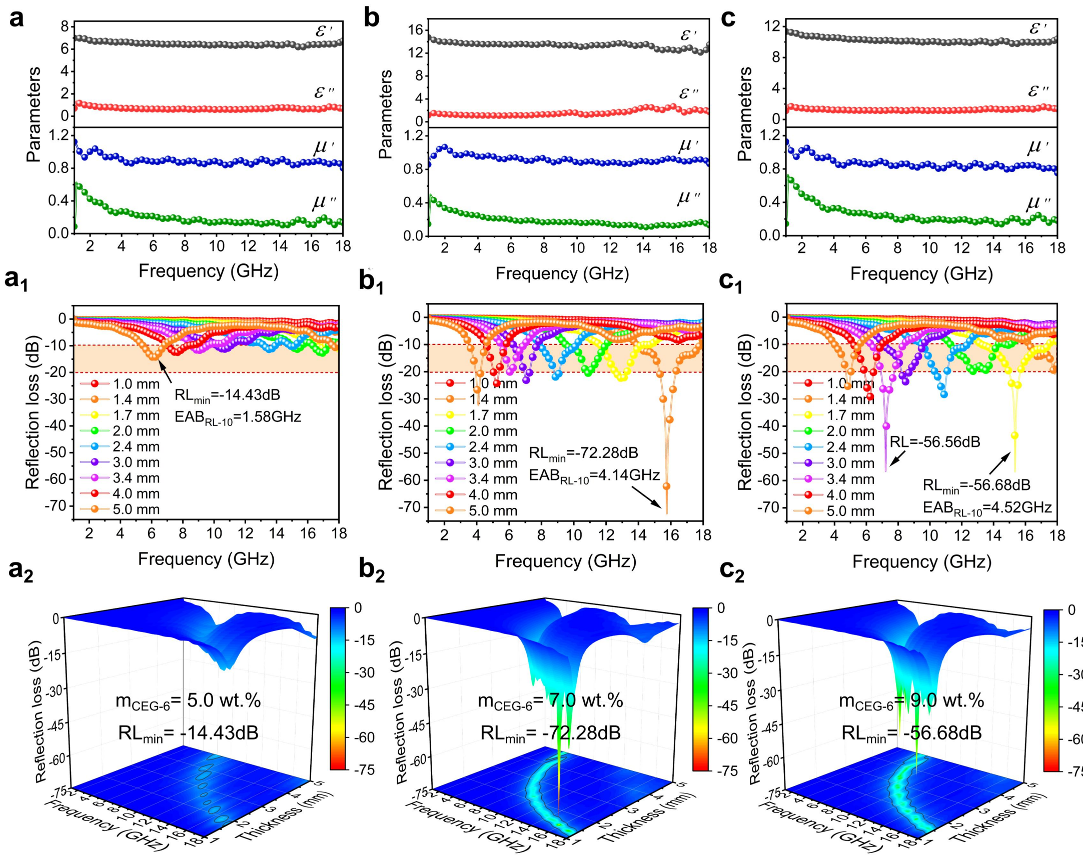

Supplementary Figure 25. EMW absorption performance of CEG-6 at different load amounts. **a**, **a_1**, **a_2** EMW absorption performance of CEG-6 at 5.0 wt% filler loading. **b**, **b_1**, **b_2** EMW absorption performance of CEG-6 at 7.0 wt% filler loading. **c**, **c_1**, **c_2** EMW absorption performance of CEG-6 at 9.0 wt% filler loading.

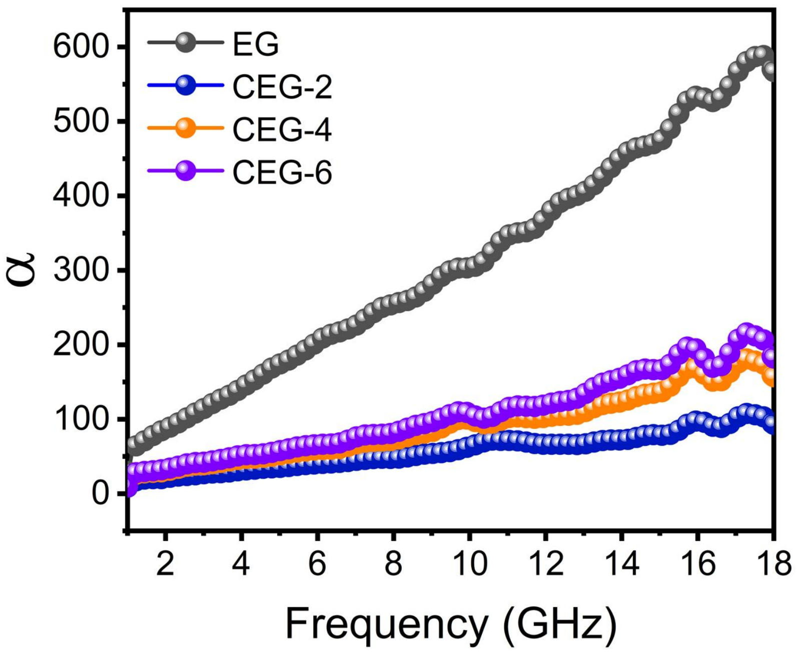

Supplementary Figure 26. Attenuation constant (α) of EG, CEG-2, CEG-4 and CEG-6.

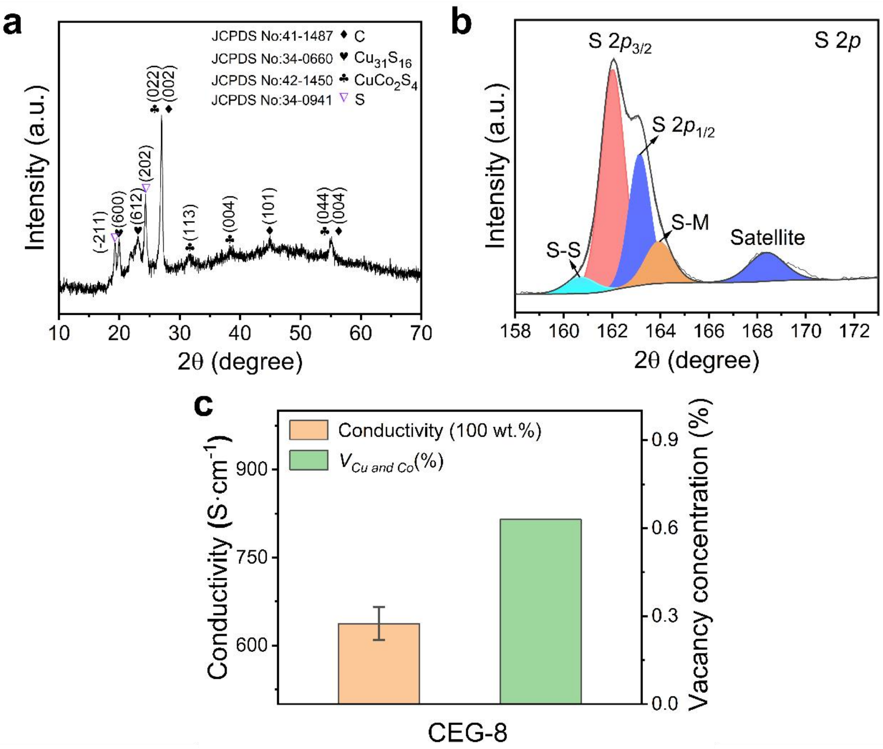

Supplementary Figure 27. Characterization of CEG-8. a XRD pattern of CEG-8. **b** S 2*p* XPS spectra of CEG-8. **c** Conductivity and vacancy concentration of CEG-8, the error bars represent standard deviations were estimated by two experiments.

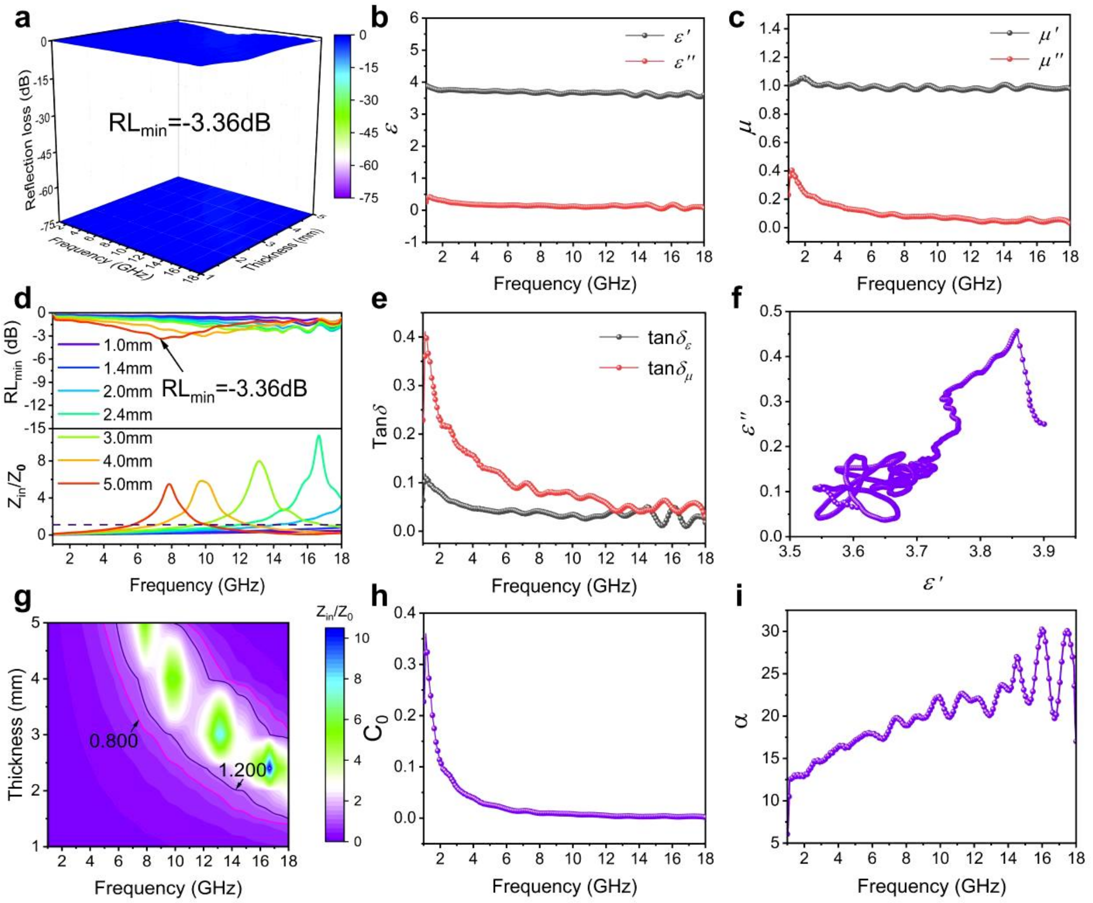

Supplementary Figure 28. EMW absorption performance of CEG-8. a, **d** RL maps of CEG-8. **b**, **c**, **e** Electromagnetic parameters (complex permittivity ($\varepsilon_r = \varepsilon' - j\varepsilon''$)and permeability ($\mu_r = \mu' - j\mu''$)) of CEG-8. **f** Cole-Cole curve of CEG-8. **g** $|Z_{in}/Z_0|$ values of CEG-8. **h** C_0 of CEG-8. **i** Attenuation constant of CEG-8.

Simulation of Electromagnetic Response

Computer Simulation Technology Microwave Studio (CST MWS) software was performed to investigate electromagnetic response of CEG by frequency domain simulation when the electromagnetic (EM) wave of 1-18 GHz was incident. In this simulation, the tested model consists of a fixed square area of 200 × 200 mm^2(a = 200 mm) were proposed. As shown in Supplementary Figure 29, the left layer is EM

absorber layer with the thickness of 1.4 mm (d_1 = 1.4 mm), and the right layer is perfect electric conductor (PEC) layer with the thickness of 1.4mm (d_2 = 1.4 mm). The boundary conditions were applied with the electric field along y direction and the magnetic field along z direction. The open boundary conditions were used in all directions. But different monitoring frequencies were set for different samples. The radar cross section (RCS) of microwave absorbers was simulated based on far-field response. The scattering direction was determined by θ and φ.

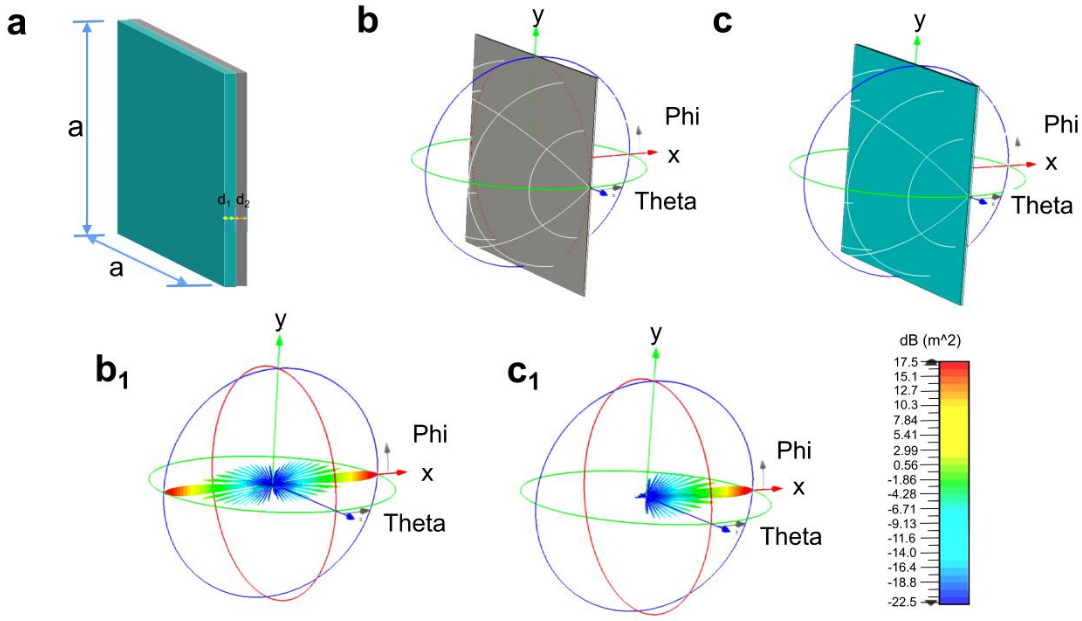

Supplementary Figure 29. Simulation of Electromagnetic Response.

a Construction of computational models. b The model of PEC for CST, **c** The model of CuCo2S4@EG. **b1, c1** PEC and $CuCo_2S_4$@EG for CST simulation.

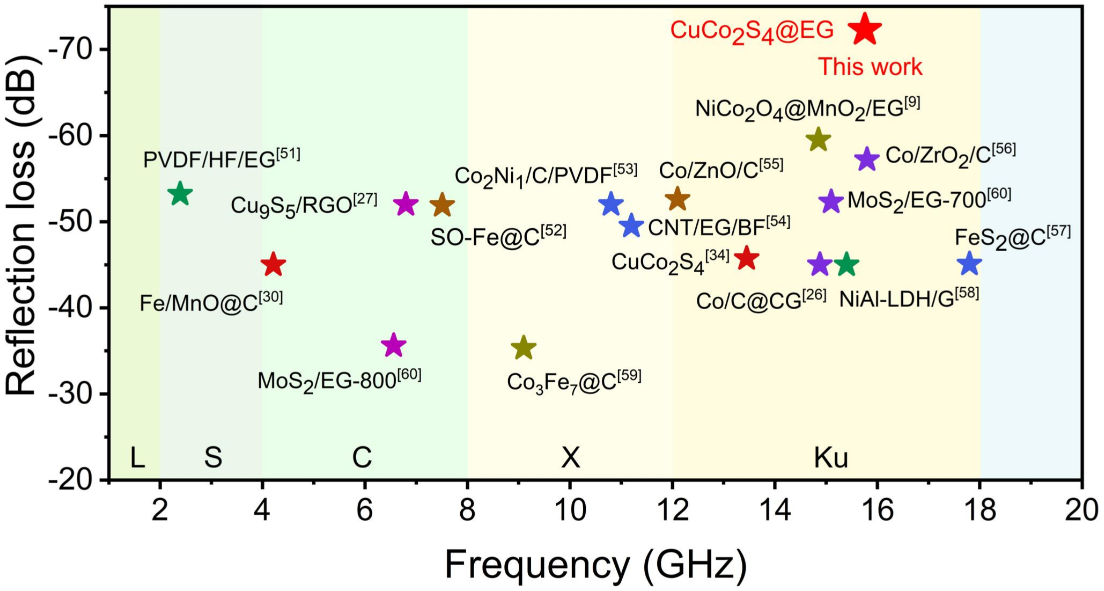

Supplementary Figure 30. Comparison with previously reported similar carbon-based EMW absorbing materials.

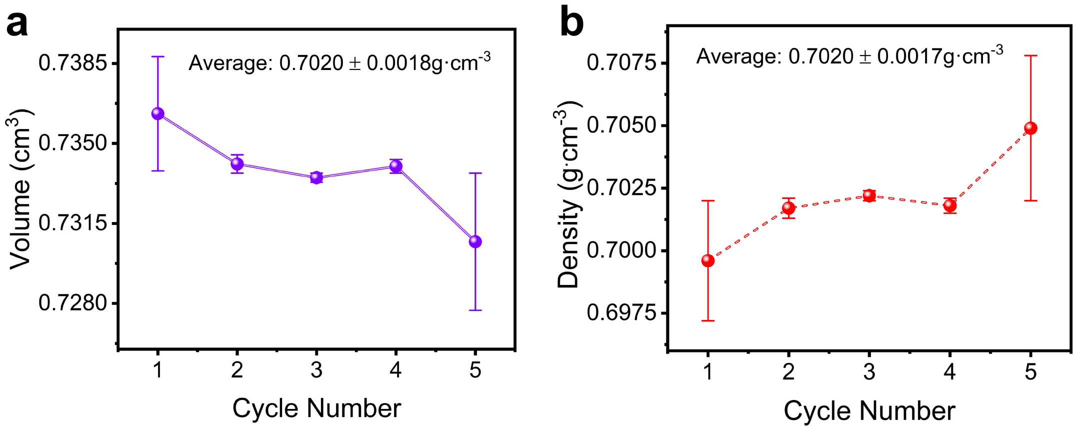

Supplementary Figure 31. True density of CEG-6 samples. a Volume of CEG-6. **b** Density of CEG-6. The error bars represent standard deviations were estimated by ten experiments.

Supplementary Table 1. Comparison with previously reported EG and similar carbon materials.

Ref.no.	Method	(S cm^{-1})	K (W m^{-1} k^{-1})
[4]	Oxidation and inserting in HNO_3 and H_2SO_4 (48-72 h)	300	-
[5]	Oxidation and inserting in HNO_3 and H_2SO_4 (2-3 days)	386	112
[6]	Oxidation and inserting in H_2SO_4, H_2O_2 and $KMnO_4$ annealing at 800℃ for 5 min	-	237
[7]	Sonication in alcoholwater mixture for 20 h annealing at 1060℃ for 2 h	850	220
[8]	Chemical vapor deposition	1136	-
[9]	Ball mill with oxalic acid (12 h) and dispersed in NMP annealing at 600℃ (2 h)	277	-
[10]	Oxidation and inserting in H_2SO_4, $KMnO_4$ and H_2O_2 (1.35h) annealing at 2000℃ for 1h	1000	1100
[11-12]	Oxidation and inserting in H_2SO_4 and HNO_3 (10-15h) annealing at 800-900℃ for 10-20s	1000	-
[13]	Stirring and sonication for 0.5 h, annealing at 450℃ (5 min) mechanical compression at 20 MPa (5min)	1467	348
	**This work**	1012	583

Supplementary Table 2. EXAFS fitting parameters at the Cu and Co K-edge for various samples.

Sample	Shell	*CN*[a]	*R*(Å)[b]	σ^2(10^{-3}Å^2)[c]	ΔE_0(eV)[d]	*R* factor
**Cu foil**	Cu-Cu	12*	2.56±0.004	8.19±0.22	4.87±0.71	0.0039
**CuO**	Cu-O1	2.9±0.3	1.96±0.009	2.28±1.37	11.80±0.93	0.0143
	Cu-O2	2.8±0.5	2.87±0.021			
**Cu CEG-6**	Cu-S	3.2±0.5	2.28±0.017	12.09±2.76	3.64±1.68	0.0112
**Co foil**	Co-Co	12*	2.51±0.005	7.34±0.28	8.85±0.70	0.0045
**Co_3O_4**	Co-O	3*	1.91±0.005	1.41±0.39	12.61±0.59	0.0036
	Co-Co1	3.6±0.4	2.87±0.003	4.37±1.01		
	Co-Co2	2.9±0.2	3.36±0.005	1.41±0.39		
**Co CEG-6**	Co-S	2.9±0.3	2.26±0.023	0.003±0.00	3.85±2.77	0.013

[a]*CN*, coordination number; [b]*R*, distance between absorber and backscatter atoms; [c]σ^2, Debye-Waller factor to account for both thermal and structural disorders; [d]ΔE_0, inner potential correction; *R* factor indicates the goodness of the fit. S_0^2 was fixed to 0.85. A reasonable range of EXAFS fitting parameters: $0.600 < S_0^2 < 1.000$; $CN > 0$; $\sigma^2 > 0$ Å^2; $|\Delta E_0| < 15$ eV; *R* factor < 0.02. Fitting range: 3.0 ≤ k (/Å) ≤ 10.0 and 1≤ R (Å) ≤ 3.0 (Co foil); 3.0 ≤ k (/Å) ≤ 12and 1.0 ≤ R (Å) ≤ 3.5 (Co_3O_4); 3.0≤k (/Å) ≤ 9 and 1.0 ≤ R (Å) ≤ 2.2(Co sample).

Supplementary Table 3. Comparison with the heat dissipation properties previously reported for similar carbon materials.

Material	Temperature (℃)	Cooling time (s)	Ref.no.
EG/LLDPE-3D network composites	90 to 20	100	[14]
APPW/EG composites	70 to 20	25	[15]
Co@C/CG aerogels	87.2 to 25.7	15	[16]
EG/BN-103 composites	182.2 to 37.6	16	[17]
EG	83.7 to 36.4	2	[18]
**This work**	199.7 to 35.8	10	

Supplementary References

[1] Kresse, G. et al. From ultrasoft pseudopotentials to the projector augmented-wave method. *Physical Review B*. **59**, 1758-1775 (1999).

[2] Perdew, J. P. et al. Generalized Gradient Approximation Made Simple. *Physical Review Letters*. **77**, 3865-3868 (1996).

[3] Grimme, S. et al. A consistent and accurate ab initio parametrization of density functional dispersion correction (DFT-D) for the 94 elements H-Pu. *Journal of Chemical Physics*. **132**, 154104 (2010).

[4] Song, W. L. et al. Magnetic and conductive graphene papers toward thin layers of effective electromagnetic shielding. *J. Mater. Chem. A*. **3**, 2097 (2015).

[5] Liang, Q. Z. et al. A Three-Dimensional Vertically Aligned Functionalized Multilayer Graphene Architecture: An Approach for Graphene-Based Thermal Interfacial Materials. *ACS Nano*. **5**, 2392-2401 (2011).

[6] Zhao, X. M. et al. Bioinspired modified graphite film with superb mechanical and thermoconductive properties. *Carbon*. **181**, 40-47 (2021).

[7] Hou, Z. L. et al. Flexible Graphene−Graphene Composites of Superior Thermal and Electrical Transport Properties. *ACS Appl. Mater. Interfaces*. **6**, 15026-15032 (2014).

[8] Zhang, L. et al. Preparation and Characterization of Graphene Paper for Electromagnetic Inter-ference Shielding. *Carbon*. **82**, 353 (2015).

[9] Lin, T. Q. et al. Facile and economical exfoliation of graphite for mass production of high-quality graphene sheets, *J. Mater. Chem. A*. **1**, 500 (2013).

[10] Chen, B. et al. Ultrathin Flexible Graphene Film: An Excellent Thermal Conducting Material with Effi cient EMI Shielding. *Adv. Funct. Mater.* **24**, 4542-4548 (2014).

[11] Dhakate, S. R. et al. Influence of Expanded Graphite Particle Size on the Properties of Composite Bipolar Plates for Fuel Cell Application. *Energy Fuels*. **23**, 934-941 (2009).

[12] Dhakate, S. R. et al. Development and Characterization of Expanded

Graphite-Based Nanocomposite as Bipolar Plate for Polymer Electrolyte Membrane Fuel Cells (PEMFCs). *Energy Fuels*. **22**, 3329-3334 (2008).

[13] Liu, Y. H. et al. Graphene enhanced flexible expanded graphite film with high electric, thermal conductivities and EMI shielding at low content. *Carbon*. **133**, 435 (2018).

[14] Wei, B. J. et al. Polymer Composites with Expanded Graphite Network with Superior Thermal Conductivity and Electromagnetic Interference Shielding Performance. *Chem Eng J*. **404**, 126437 (2021).

[15] Xie, Y. P. et al. Highly thermally conductive and superior electromagnetic interference shielding composites via in situ microwave-assisted reduction/exfoliation of expandable graphite. C*ompos part A-APPL S*. **149**, 106517 (2021).

[16] Xu, J. et al. Lightweight, Fire-Retardant, and Anti-Compressed Honeycombed-Like Carbon Aerogels for Thermal Management and High-Efficiency Electromagnetic Absorbing Properties, *Small*. **17**, 2102032 (2021).

[17] Nie, Z. G. et al. Layered-structure N-doped expanded-graphite/boron nitride composites towards high performance of microwave absorption. *J Mater Sci Technol*. **113**, 71-81 (2022).

[18] Wei, Q. et al. High-performance expanded graphite from flake graphite by microwave-assisted chemical intercalation process. *J Ind Eng Chem*. **122**, 562-572 (2023).
